# A single-dose mRNA vaccine induces potent and long-lasting humoral and cellular immunity against the varicella-zoster virus in a murine model

**DOI:** 10.3389/fimmu.2026.1771359

**Published:** 2026-03-23

**Authors:** Wenyu Cheng, Ning Ren, Li Xie, Wenjin Zhou, Rui Quan, Heng Wei

**Affiliations:** Chengdu Kanghua Biological Products Co., Ltd., Chengdu, Sichuan, China

**Keywords:** CD4^+^ memory T cells, glycoprotein E, heterologous immunization, humoral and cellular immunity, mRNA vaccine, varicella-zoster virus

## Abstract

**Introduction:**

Herpes zoster is an infectious disease caused by the varicella-zoster virus (VZV). In adults, the reactivation of VZV can lead to severe neuralgia and skin rashes. Although the licensed vaccines are available, their associated adverse reactions and the shortage of required adjuvants necessitate the development of novel VZV vaccines.

**Methods:**

We developed a novel VZV mRNA vaccine candidate (named as KH014) containing sequence-optimized mRNAs encoding full-length glycoprotein E encapsulated in an ionizable lipid nanoparticle. Its immunogenicity was evaluated in BALB/c mice receiving single-dose, two-dose, or heterologous prime-boost regimens in comparison with the licensed adjuvanted subunit vaccine Shingrix®. Humoral responses were assessed by ELISA and microneutralization assays; cellular immunity was characterized by ELISpot, intracellular cytokine staining, and memory T-cell phenotyping via flow cytometry.

**Results:**

In mice, immunization with either a single-dose or two-dose of KH014 elicited superior VZV-specific humoral and cellular immune responses compared to the licensed vaccine Shingrix^®^. Furthermore, the antigen-specific CD4^+^ T-cell responses elicited by a single KH014 were significantly higher and persisted for at least 10 weeks, underscoring the capacity of KH014 to induce durable protective immunity against VZV. Heterologous KH014 prime/Shingrix^®^ boost further enhanced cellular responses, including polyfunctional CD4⁺ and CD8⁺ T cells, and induced favorable central and effector memory T-cell profiles.

**Conclusion:**

The KH014 mRNA vaccine candidate induces robust and durable humoral and cellular immunity against VZV in mice, with a single dose sufficient to elicit T-cell responses superior to those of two-dose Shingrix^®^. These findings support further development of KH014 as a potential single-dose vaccine and inform heterologous immunization strategies to optimize protective efficacy against herpes zoster.

## 1 Introduction

Varicella-zoster virus (VZV) establishes latency after primary infection, with reactivation causing herpes zoster (HZ) and postherpetic neuralgia (PHN) mainly in older adults and immunocompromised individuals, severely impacting quality of life (1–4). Globally, the cumulative incidence of HZ is 2.9 to 19.5 per 1000 population, and is higher for females (3.22-11.2 per 1000 population) than males (2.44-8.0 per 1000 population) (5). In China, it has been estimated that the incidence rate is 2.9 to 5.8 per 1000 annually, which incurs billions in cost annually to the healthcare system and to society through loss of productivity (6). Notably, incidence and PHN risk rise markedly after age 50-60, correlating with an age-related decline in cell-mediated immunity (CMI) (1, 4). Thus, the induction efficient VZV-specific CMI responses are critical for the prevention and control of initial VZV infection, as well as the reactivation of latent infection (3). Vaccination is the main strategy for the prevention of HZ. Currently, two main types of VZV vaccines have been approved, including live attenuated VZV (e.g., ZOSTAVAX^®^ manufactured by Merck Sharp & Dohme, USA and Ganwei^®^ manufactured by Changchun BCHT Biotechnology Co. Ltd., China) and recombinant VZV glycoprotein E (Shingrix^®^ manufactured by GSK) (7). However, live attenuated vaccines show modest or declining efficacy in the elderly (7–9). The two-dose intramuscular administration of Shingrix^®^ (adjuvanted with AS01B) demonstrates greater than 90% vaccine efficacy but shows severe systemic reactions within 7 days post-vaccination and depends on a limited adjuvant supply (10–14). These challenges highlight the necessity for novel, improved VZV vaccines.

Studies investigating the immune mechanisms underlying Shingrix^®^ efficacy indicate that robust VZV-specific CD4^+^ T-cell responses are key determinants of protection (10). RNA technology has unique advantages in activating innate immune and inducing CMI responses without the addition of adjuvants (14). Therefore, the mRNA platform provides a new strategy for the development of VZV vaccines. It is well known that VZV gE is the most abundant and highly immunogenic VZV antigen with conserved T cell epitopes (15). Research from two independent studies demonstrates that full-length gE mRNA-LNP vaccines elicit humoral responses comparable to Shingrix^®^ and induce stronger T cell responses in mice and rhesus macaques (11, 16, 17). Comparative analysis of several gE-based mRNA vaccine constructs including truncated, soluble, and epitope-enriched variants, as well as constructs co-encoding IE62, the full-length gE construct CVG206 elicited significantly superior gE-specific IgG titers and more robust T cell responses (18). A novel VZV mRNA vaccine candidate mRNA-1468 (featuring truncated gE with a Y569A mutation) from Moderna has been tested and was shown to induce comparable levels of humoral and cellular immune responses to Shingrix^®^ (10). Building on these findings, a novel full-length optimized gE mRNA vaccine (named as ZOSAL) developed by Lin Ang group demonstrated superior immunogenicity and safety in multiple aspects over Shingrix^®^, especially in the induction of strong T-cell immunity (16). This suggested a high potentiality of mRNA technology in the development of next-generation VZV vaccines.

An ideal VZV vaccine would confer effective protection through a single-dose inoculation, thereby shortening the period of risk and improving vaccine acceptance (19). Most candidate mRNA vaccines, however, still require two doses. Here, we developed KH014, a full-length gE mRNA encapsulated in ionizable LNPs. Following its promising immunogenicity with two doses, we evaluated a single-dose regimen. Compared to Shingrix^®^, a single dose of KH014 elicited superior VZV-specific CMI and long-term immunity. We further assessed heterologous prime-boost strategies with KH014 and Shingrix^®^, providing a framework for improved sequential immunization strategies.

## 2 Materials and methods

### 2.1 Ethics statement

All mice were handled in accordance with the Good Animal Practice Requirements of the Animal Ethics Procedures and Guidelines of the People’s Republic of China, and the protocol was reviewed and approved by the Animal Ethics Committee of Chengdu Kanghua Biological Products Co. Ltd (Permit No. AEC-CDKH2025-0014).

### 2.2 Cloning and mRNA preparation

The coding sequence (CDS) of gE derived from the sequence of the VZV vOKa strain was codon optimized and synthesized, then cloned into a plasmid vector with backbone sequence elements of a high copy origin of replication, a kanamycin resistance gene, a T7 promoter, 5′- and 3′ UTR, and a 100-nucleotide poly(A) tail. The plasmid DNAs were sequence verified, linearized by digestion with *Bsp*Q I downstream of poly(A) and purified by DNA Clean Beads (N411-01; Vazyme Biotech Co., Ltd., Nanjing, China). Following linearization, gE mRNA (named as KH014) sequence was synthesized with a T7 High Yield RNA Transcription Kit (TR101-01; Vazyme Biotech Co., Ltd., Nanjing, China) and N1-methyl-pseudouridine-5’-triphosphate (m1Ψ) instead of Uridine-5’-Triphosphate (UTP). Synthesized mRNA was further capped using a Vaccinia Capping System (DD4109-01; Vazyme Biotech Co., Ltd., Nanjing, China) and purified via RNA Clean Beads (N412-01; Vazyme Biotech Co., Ltd., Nanjing, China). The length and integrity of gE mRNA was analyzed using capillary electrophoresis. RNA characteristics including concentration, sequence length, and integrity were assessed by ultraviolet spectrophotometer, agarose gel electrophoresis and capillary electrophoresis, respectively.

### 2.3 Preparation of vaccines

For the preparation of LNP, lipids were dissolved in ethanol at a molar ratio of 50:10:38.5:1.5 (SM-102: DSPC: cholesterol: PEG2000), and mRNA-gE-LNPs were dissolved at a charge ratio of N/P=6 in a 50 mM sodium citrate buffer (pH=4) solution. LNPs were formulated using a microfluidic mixer (Micro & Nano Technology, China) by mixing the aqueous and organic solutions at a ratio of 3:1. The LNP solution was concentrated by ultrafiltration using the Consieve TFF Ultrafiltration Cassettes (UFCLA0300001P; Hangzhou Cobetter Filtration Equipment Co. Ltd, Hangzhou, China), according to the manufacturer’s instructions. The mRNA-LNP was dispensed into appropriate cryoprotectant in vials, and then were lyophilized. LNP formulations were tested for particle size by Dynamic Light Scattering, mRNA encapsulation and concentration by RiboGreen Assay, RNA integrity by capillary electrophoresis (Agilent Fragment Analyzer).

### 2.4 *In vitro* expression assay

*In vitro* expression of KH014 mRNA-LNP was assessed by ELISA kit (RAS-A185; Acrobiosystems Inc., Beijing, China). HepG2 cells were seeded into 12-well culture plates and cultured in Dulbecco’s Modified Eagle Medium (DMEM; Gibco; Thermo Fisher Scientific, Inc., MA, USA) supplemented with 10% fetal bovine serum (FBS; Gibco; Thermo Fisher Scientific, Inc., MA, USA) and 1% penicillin-streptomycin at 37˚C in the presence of 5% CO_2_. Once confluency was reached approximately 80%, cells were transfected with LNPs containing 2 μg KH014 mRNA or empty LNP. After 24 h of incubation at 37 °C, the supernatants and cells were harvested together, and then lysed by three cycles of freeze-thawing. The gE protein expression levels in the supernatant clarified by centrifugation at 1,000 g/rpm for 10 min were detected using ELISA kit according to the manufacturer’s protocol.

### 2.5 Animal immunizations, blood collection and tissue harvest

Female specific pathogen-free BALB/c mice between 6 and 8 weeks of age were purchased from Chengdu GemPharmatech Co. Ltd. and housed in individually ventilated cages (IVCs). For mRNA immunization groups, mice were received intramuscular injections of single or two doses of KH014 on days 0 and/or 21. Each dose contained 1μg, 5μg, 10 μg, or 20 μg of mRNA for LNPs. Additionally, the positive group was immunized intramuscularly with two doses of 0.1 human dose of Shingrix^®^ at a 3-week interval (17, 20). Serum samples were collected from the submandibular (facial) vein of mice for antibody response analysis. Mice were euthanized by cervical dislocation at the indicated time points after the final vaccination. Spleens were collected and processed to obtain single-cell suspension for cellular immune responses analysis.

### 2.6 Enzyme-linked immunosorbent assay of antibody titers

gE proteins (GLE-V52H3; Acrobiosystems Inc., Beijing, China) were coated into 96-well plates (42592; Corning Incorporated Life Sciences, Shanghai, China) at a concentration of 0.1 μg/well and incubated overnight at 4 °C. The plates were washed three times with PBS containing 0.1% Tween-20 (PBST) and blocked for two hours at room temperature (RT) with 2% bovine serum albumin (BSA). Serially diluted serum samples (ranging from 625,000 to 80,000,000) were added and incubated for 2 h at room temperature. After triple washing with PBST solution, goat anti-mouse IgG HRP-conjugated antibody (Abcam, ab205719, 1:5000 dilution) was added and incubated at 37 °C for 40 min. Then, the plates were washed with PBST and TMB substrate was added to each well. The reaction was stopped with sulfuric acid, and absorbance was measured at 450/620 nm using a microplate reader. Endpoint titers were defined as the highest serum dilution yielding an optical density (OD) > 2.1× background.

### 2.7 *In vitro* microneutralization assay

Vazyme Testing Technology Co., Ltd. (Nanjing, China) conducted microneutralization assays (21). MRC-5 cells were seeded in 96-well plates and incubated overnight (37 °C, 5% CO_2_). Heat-inactivated sera (56 °C, 30 min) underwent serial 3-fold dilutions from 1:30, generating eight concentrations in duplicate. Diluted virus was added to samples/viral controls, neutralized (1 h, 37 °C), then transferred to cell monolayers with controls (50 μL/well, duplicate). After 2 h incubation, medium was replaced. Following 48 h incubation, cells were fixed, stained with fluorescent antibodies, and analyzed using CTL. Neutralization activity (ID_50_) was calculated using the Reed-Muench method by comparing infected cells to virus controls, with ≥30 as positive cutoff (21).

### 2.8 IFN-γ/IL-2 fluorospot assay

For the ELISpot assay, splenocytes were isolated and resuspended in RPMI-1640 medium supplemented with 10% FBS (Gibco; Thermo Fisher Scientific, Inc., MA, USA) and penicillin-streptomycin (15140122; Gibco; Thermo Fisher Scientific, Inc., MA, USA) at a final concentration of 10^7^ cells/mL. IL-2 and IFN-γ positive splenocytes were performed according to the manufacturer’s instructions for the ELISpot assay (MabTech AB, Stockholm, Sweden). Briefly, splenocytes (3×10^5^ cells/well) were seeded in the IFN-γ/IL-2 antibodies precoated plates and stimulated at 37 °C for 48 h using a protein gE peptide pool at a concentration of 2 μg/mL. In the positive control well, PMA + ionomycin was used as a stimulus. After five times washing, detection antibodies were added and incubated at room temperature for 2 h. Finally, fluorophore-conjugated reagents and fluorescence enhancer were added to each well. Plates were read and analyzed in an FluoroSpot reader system (iSpot Spectrum, AID, Strassberg, Germany).

### 2.9 Flow cytometry

Splenocyte suspensions were incubated in a 96-well plate (10^6^ cells/well) with 200 μL of RPMI 1640 medium supplemented with 10% FBS, 1% penicillin-streptomycin, and 1× protein transport inhibitor cocktail (Invitrogen). The VZV gE peptide mix (2 µg/mL final concentration) or concanavalin A was added to each well and incubated for 5 h at 37 °C under 5% CO_2_. During the final 2 h, 25 µL each of GolgiPlug and GolgiStop were added to all samples. After centrifugation at 4 °C for 10 min, cells were stained using the LIVE/DEAD fixable violet dead cell stain kit (Invitrogen) following the manufacturer’s instructions. Following two washes, Fc receptors were blocked with anti-mouse CD16/32 antibody (553142; BD Biosciences) for 10 min. Subsequently, cells were stained with surface antibodies including FITC anti-mouse CD3 antibody (100204, Biolegend), APC anti-mouse CD4 antibody (100412, Biolegend), and Brilliant Violet 650 anti-mouse CD8a antibody (100742, Biolegend) for 30 min. After surface staining, cells were fixed and permeabilized using Cytofix/Cytoperm Soln Kit (554714, BD Biosciences) according to the manufacturer’s instructions. Intracellular cytokine expression was assessed using Brilliant Violet 421 anti-mouse IFN-γ (505830; BioLegend), PE anti-mouse TNF-α (506306; BioLegend), PerCP/Cyanine5.5 anti-mouse IL-4 (504123; BioLegend) and PE/Cyanine7 anti-mouse IL-2 (503832; BioLegend). For the analysis of memory T cells, the cells were incubated with PerCP/Cyanine5.5 anti-mouse CD3 antibody (100218, Biolegend), FITC anti-mouse CD4 antibody (100204, Biolegend), PE anti-mouse CD62L antibody (161204, Biolegend), and APC anti-mouse CD44 antibody (103012, Biolegend) for 30 min. Intracellular IL-2 and IFN-γ expression in memory T cells was detected using Brilliant Violet 421 anti-mouse IFN-γ (505830; BioLegend) and PE/Cyanine7 anti-mouse IL-2 (503832; BioLegend). Cells were washed with intracellular staining perm/wash buffer and resuspended in 1× PBS for flow cytometry analysis (CytoFLEX, Beckman Coulter).

### 2.10 Histological staining

Tissue samples were fixed in 10% formalin, embedded in paraffin, and sectioned at 5 μm. Sections were stained with hematoxylin and eosin (HE) and scanned using 3DHISTECH.

### 2.11 Haematology analysis

For hematology parameters, blood was collected in potassium EDTA tubes and analyzed using an Advia 120 Haematology Analyser for the determination of white blood cell count, red blood cell count, platelet count, neutrophil count, percentage of lymphocyte, percentage of neutrophile, percentage of monocyte, percentage of eosinophile, and percentage of eosinophil.

### 2.12 Statistical analysis

Data are presented as means ± SD. One-way or two-way analysis of variance (ANOVA) was applied for multiple comparisons, and Student’s t-test for single comparisons. Statistical analyses were performed using GraphPad Prism 9.5.0 (GraphPad Software Inc., La Jolla, CA, USA).

## 3 Results

### 3.1 Preparation and characterization of KH014 mRNA vaccine

Since glycoprotein E (gE) is a dominant immunogen of VZV and has been widely used as a key antigen candidate for VZV vaccine development, the KH014 mRNA vaccine was designed to encode the full-length gE antigen. Using algorithms such as LinearDesign and GenScript, a codon-optimized gE mRNA sequence was generated based on the wild-type VZV Oka strain (GenBank: AY253715.1). The optimized gE mRNA was synthesized via *in vitro* transcription (IVT) incorporating N^1^-methylpseudouridine (m^1^Ψ) modification. After transcription, a 5’ cap-1 structure was enzymatically added to produce fully mature mRNA. The IVT products and capped mRNA were verified using agarose gel electrophoresis (**Supplementary Figure 1A**), revealing a single band and confirming good purity. Further analysis by capillary electrophoresis of RNA revealed a single peak at 2100 nt (**Supplementary Figure 1C**), indicating its integrity. To evaluate *in vitro* expression, naked and LNP-encapsulated gE mRNAs were transfected into HepG2 cells. Western blotting revealed a distinct band at ~60 kDa in both cell lysates and supernatants (**Supplementary Figure 1B**). As expected, ELISA detected a higher protein expression level from LNP-encapsulated mRNA in the cell lysates (**Supplementary Figure 1F**), suggesting the encoded gE protein can be secreted outside transfected cells. Then, the quality control of KH014 mRNA vaccine was conducted by examining the size distribution and zeta potential with dynamic light scattering. We discovered that KH014 mRNA vaccine displayed a good uniformity of the particle sizes (~94 nm) (**Supplementary Figure 1D**), polydispersity index (0.16) (**Supplementary Figure 1E**). Zeta-potential measurements indicated that KH014 had a surface charge of -3.89 (**Supplementary Figure 1E**). By calculating the ratio between the quality of detected mRNA and the input dose, the encapsulation efficiency of the vaccine was obtained, and the result was 94.6% (**Supplementary Figure 1E**).

### 3.2 KH014 mRNA vaccine induces VZV-specific humoral immune responses

**Figure 1:**
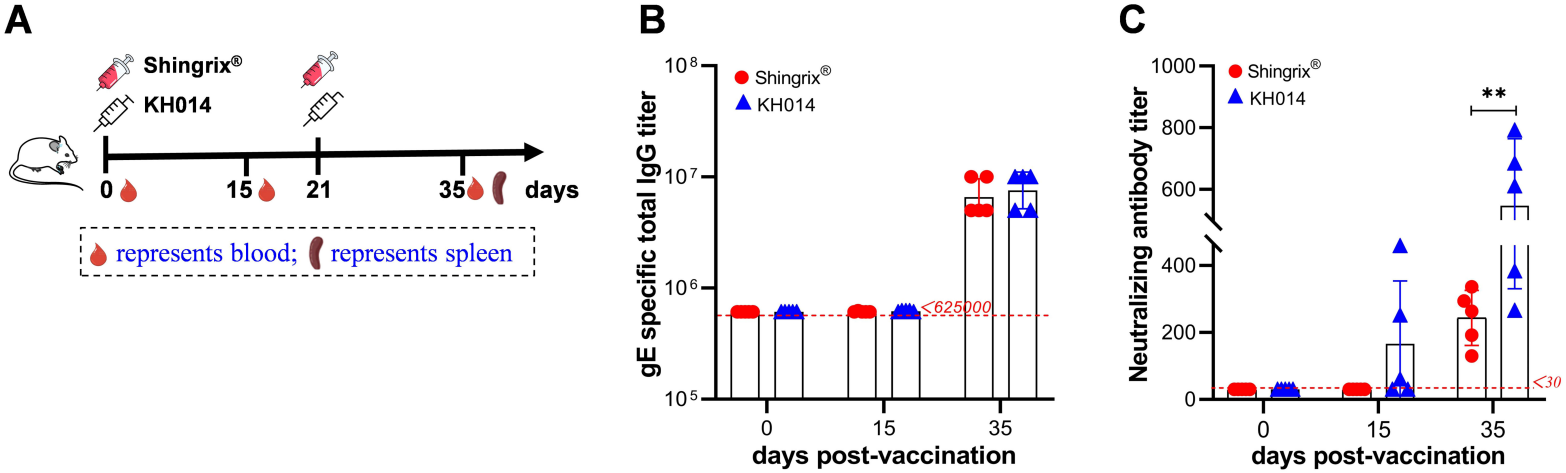
KH014 mRNA vaccine induces robust antigen-specific humoral immune responses. Female BALB/c mice (n=5) received two doses of either KH014 mRNA vaccine, Shingrix^®^, or PBS at day 0 and 21, administered via intramuscular injection. Sera were collected at day 0 (pre-vaccination), 15, and 35 post-first immunization and subjected to antibody detection. **(A)** Scheme of immunization and sample collection schedule. **(B)** gE-specific antibody responses were determined by endpoint dilution ELISA. The limit of detection of the endpoint titers were set to <625000. **(C)** Neutralizing antibody titers were measured by *in vitro* microneutralization assay. The limit of detection of the neutralizing antibody titers were set to <30. Data are shown as the mean ± SD. Each symbol represents one animal. *P* values were calculated by one-way ANOVA with multiple comparison tests. **P < 0.01.

To evaluate immunogenicity and effectiveness, mice were immunized according to the scheme in **Figure 1**A with two doses of KH014 mRNA vaccine, Shingrix^®^, or PBS via intramuscular injection. The serum samples from the mice were collected at day 0 (pre-vaccination), 15, and 35 post-first immunization. Anti-gE IgG binding antibodies in the sera were quantified using ELISA. As shown in **Figures 1B**, neither vaccine elicited detectable gE-specific IgG antibodies at baseline (day 0) or day 15, but both induced substantially elevated levels by day 35. Although KH014 mRNA vaccine generated slightly higher gE-specific binding antibody levels at day 35 than Shingrix^®^, this difference was not statistically significant.

Neutralizing antibody titers are another key indicator of vaccination efficacy and important for the varicella vaccine (21). We thus measured neutralizing antibody titers using a neutralizing assay at day 0, 15, and 35 post-first immunization (**Figure 1C**). The results showed that mice immunized with KH014 mRNA vaccine produced neutralizing antibody titers significantly higher than those of the Shingrix^®^ group by 2.25-fold at day 35. Notably, at day 15, three sera from the KH014 mRNA vaccine group exhibited measurable neutralizing activity, whereas none from the Shingrix^®^ group did, suggesting that KH014 elicited faster and stronger neutralizing antibody responses.

### 3.3 KH014 activates strong cellular immune responses and CD4^+^ memory T cells

**Figure 2:**
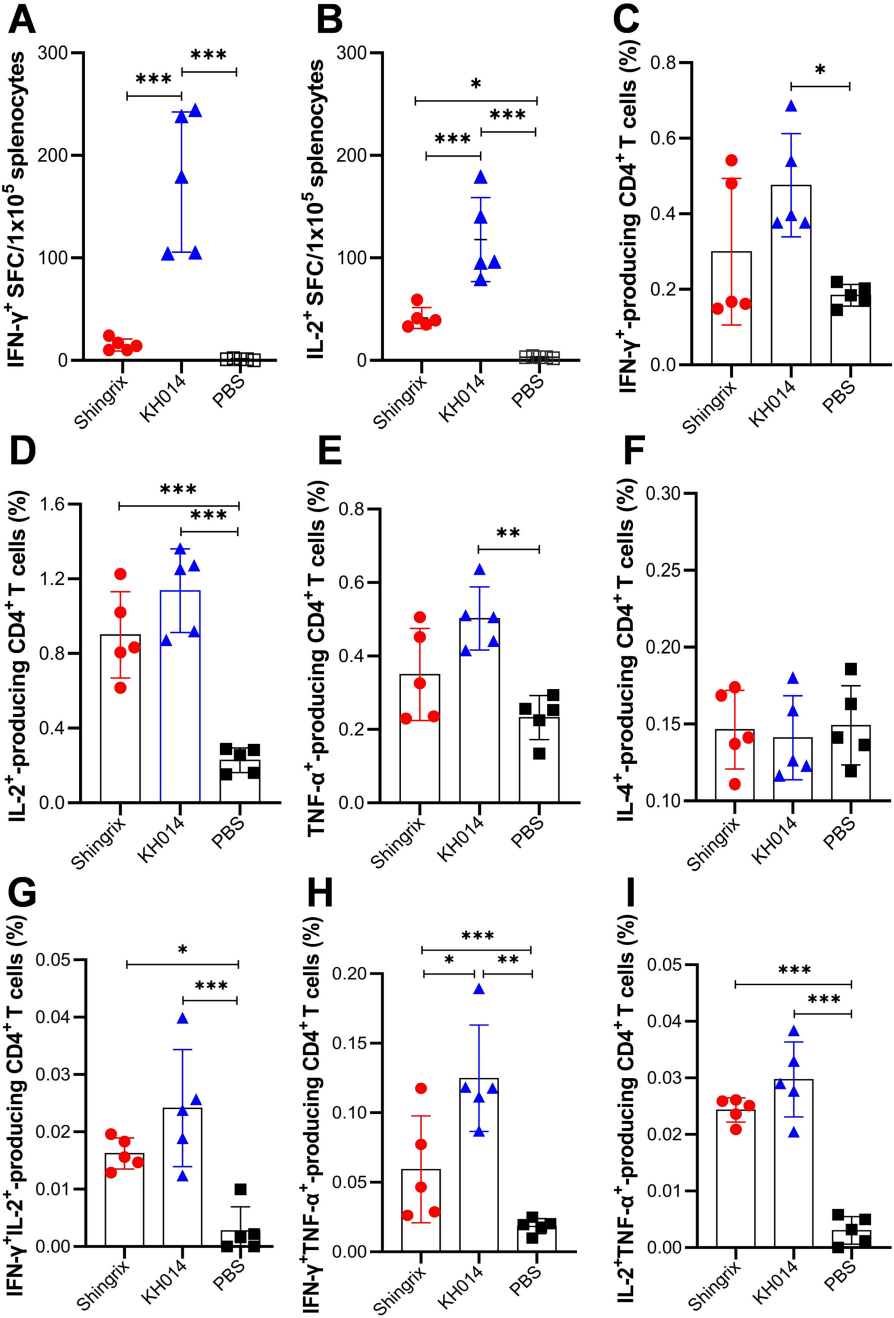
KH014 mRNA vaccine activates strong cellular immune responses. Female BALB/c mice (n=5) received two doses of either KH014 mRNA vaccine, Shingrix^®^, or PBS at day 0 and 21, administered via intramuscular injection. Two weeks after booster immunization, the mice were sacrificed, and splenocytes were collected. ELISpot assay was performed to assess IFN-γ **(A)** and IL-2 **(B)** secretion by mouse splenocytes. Percentage of antigen specific CD4^+^ T cells producing IFN-γ **(C)**, IL-2 **(D)**, TNF-α **(E)**, IL-4 **(F)**, IFN-γIL-2 **(G),** IFN-γTNF-α **(H)**, and IL-2TNF-α **(I)** from mice splenocytes were measured by intracellular cytokine staining (ICS) assay. Data are shown as the mean ± SD. Each symbol represents one animal. *P* values were calculated by one-way ANOVA with multiple comparison tests. *P < 0.05, **P < 0.01, ***P < 0.001.

Given that cellular immune responses play a key role in preventing VZV reactivation, IFN-γ and IL-2 secretion was quantified by ELISpot assay. As shown in **Figure 2A**, the number of IFN-γ-secreting cells after gE stimulation was 11.6-fold higher in KH014 mRNA group than in the Shingrix^®^ group. Consistent with IFN-γ spot, the number of IL-2 spots induced by KH014 also higher than Shingrix^®^ (**Figure 2B**). Subsequently, gE-specific T-cell responses were assessed by intracellular cytokine staining (ICS) assay to quantify IFN-γ, IL-2 and TNF-α-producing CD4^+^ (**Figures 2C–2E, 2G–2I**) and CD8^+^ T (**Supplementary Figure 2**) cells at day 14 after boost immunization. Compared with Shingrix^®^, KH014 vaccination elicited higher frequencies of Th1-type CD4^+^ T cells in spleens. For Th2-prone cytokines (IL-4), there was no significant difference between the KH014 and Shingrix^®^ groups (**Figure 2F**). Assessment of cytokines-producing CD8^+^ T cell frequencies by ICS also demonstrated higher induction levels with KH014 than with Shingrix^®^ (**Supplementary Figure 2**).

**Figure 3:**
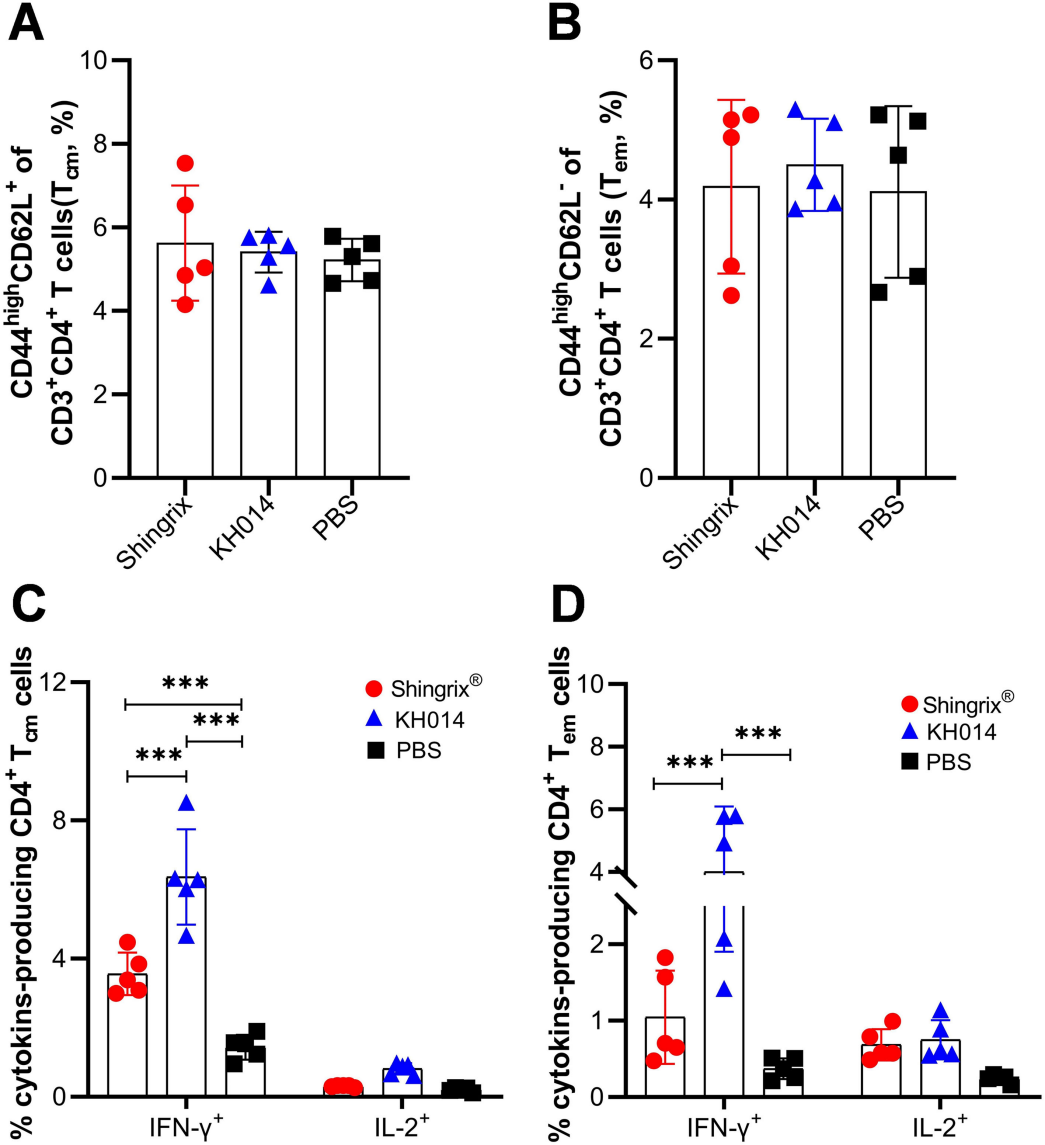
VZV-specific CD4^+^ memory T cells response induced by KH014 in mice. Female BALB/c mice (n=5) received two doses of either KH014 mRNA vaccine, Shingrix^®^, or PBS at day 0 and 21, administered via intramuscular injection. Two weeks after booster immunization, the mice were sacrificed, and splenocytes were collected. Percentage of central memory CD4^+^CD44^+^CD62L^+^ T cells (Tcm) **(A)**, effector memory CD4^+^CD44^+^CD62L^-^ T cells (Tem) **(B)**, VZV-specific Tcm **(C)** and Tem **(D)** secreting either IFN-γ or IL-2 from mice splenocytes were determined by ICS assay. Data are shown as the mean ± SD. Each symbol represents one animal. *P* values were calculated by one or two-way ANOVA with multiple comparison tests. ***P < 0.001.

Effective vaccines are expected to establish adaptive immunological memory (22). To determine whether KH014 vaccination induces long-lasting immunity, we investigated the presence of gE-specific CD4^+^ central (CD44^+^CD62L^+^) and effector (CD44^+^CD62L^-^) memory T cells in the spleens of mice, collected at day 14 after boost immunization. As shown in **Figure 3**, there were no significant differences in the proportions of CD4^+^CD44^+^CD62L^+^ and CD4^+^CD44^+^CD62L^-^ cells among all groups (**Figure 3A, B**), suggesting that the different vaccines administered to the mice had no significant effect on memory T cells. However, KH014 induced significantly higher frequencies of gE-specific central and effector memory CD4^+^ T cell responses, particularly IFN-γ-producing CD4^+^ memory T cells, compared to Shingrix^®^ (**Figure 3C, D**). These results highlight the ability of our proposed KH014 vaccine candidates to elicit a potent T cell and long-lasting immune responses against VZV in the mouse model.

### 3.4 A single dose of the KH014 elicits robust humoral and cellular immune responses in mice

**Figure 4:**
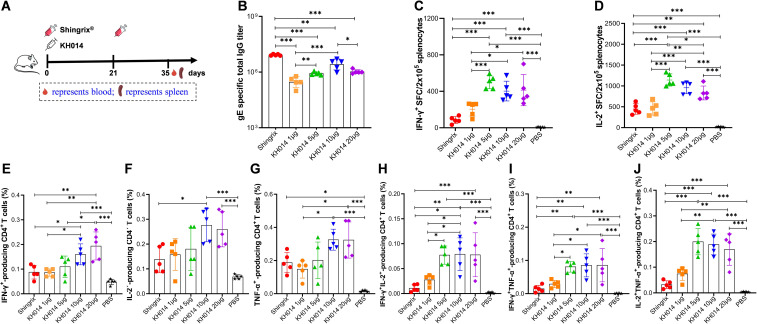
A single dose of the KH014 elicits robust humoral and cellular immune responses in mice. BALB/c mice (n=5) received a single-dose of 1 µg, 5 µg, 10 µg or 20 µg of KH014 via intramuscular injection. Control mice received two intramuscular 5-μg doses of Shingrix^®^, or PBS at day 0 and 21. On day 35, all mice were sacrificed to collect serum and splenocyte samples. **(A)** Scheme of immunization and sample collection schedule. **(B)** gE-specific antibody responses were determined by endpoint dilution ELISA. ELISpot assay was performed to assess IFN-γ **(C)** and IL-2 **(D)** secretion by mouse splenocytes. Percentage of antigen specific CD4^+^ T cells producing IFN-γ **(E)**, IL-2 **(F)**, TNF-α **(G)**, IFN-γIL-2 **(H),** IFN-γTNF-α **(I)**, and IL-2TNF-α **(J)** from mice splenocytes were measured by ICS assay. Data are shown as the mean ± SD. Each symbol represents one animal. *P* values were calculated by one-way ANOVA with multiple comparison tests. *P < 0.05, **P < 0.01, ***P < 0.001.

Given that vaccination with KH014 provided potent humoral and cellular immune responses, we investigated whether a single-dose administration of the vaccine would confer protection. BALB/c mice were vaccinated with escalating dosages (1 μg, 5 μg, 10 μg, and 20 μg) of KH014 mRNA vaccine (**Figure 4A**). As a positive control, mice received two intramuscular 5-μg doses of Shingrix^®^ on days 0 and 21. By ELISA, serum samples collected at day 35 were evaluated for gE-specific IgG. All four dosages of KH014 induced potent levels of gE-specific antibody following a single-dose immunization (**Figure 4B**). Furthermore, administration of KH014 at doses below 10 μg stimulated the production of gE-specific IgG titers in a dose-dependent manner. With regards to the cellular immune response, splenocytes were subjected to enzyme-linked immunospot (ELISpot) and intracellular cytokine staining (ICS) assays at day 35. ELISpot assays revealed that KH014 administered at dosage of 5 μg or 10 μg elicited a significantly higher level of IFN-γ or IL-2-producing T cells than Shingrix^®^ (**Figures 4C, D**). Among the various immunization dosage groups evaluated, the 5-µg mRNA dose induced the highest number of IFN-γ and IL-2 spots. Flow cytometry further demonstrated that KH014 administered at ≥ 5 µg doses elicited substantially higher proportions of CD4^+^ T cells expressing one or two cytokines than Shingrix^®^, while responses in the 1 µg group were comparable (**Figures 4E–J**). However, frequencies of cytokine-producing CD8^+^ T cells (except IL-2-producing CD8^+^ T cells) induced by all KH014 immunization groups were moderate elevated compared to Shingrix^®^-treated animals (**Supplementary Figure 3**). Collectively, these findings indicate that even a single low dose of KH014 effectively activates both humoral and gE-specific T-cell immunity, with 5 μg or 10 µg doses producing stronger responses than Shingrix^®^.

### 3.5 A single dose of KH014 induces long-term immunogenicity

**Figure 5:**
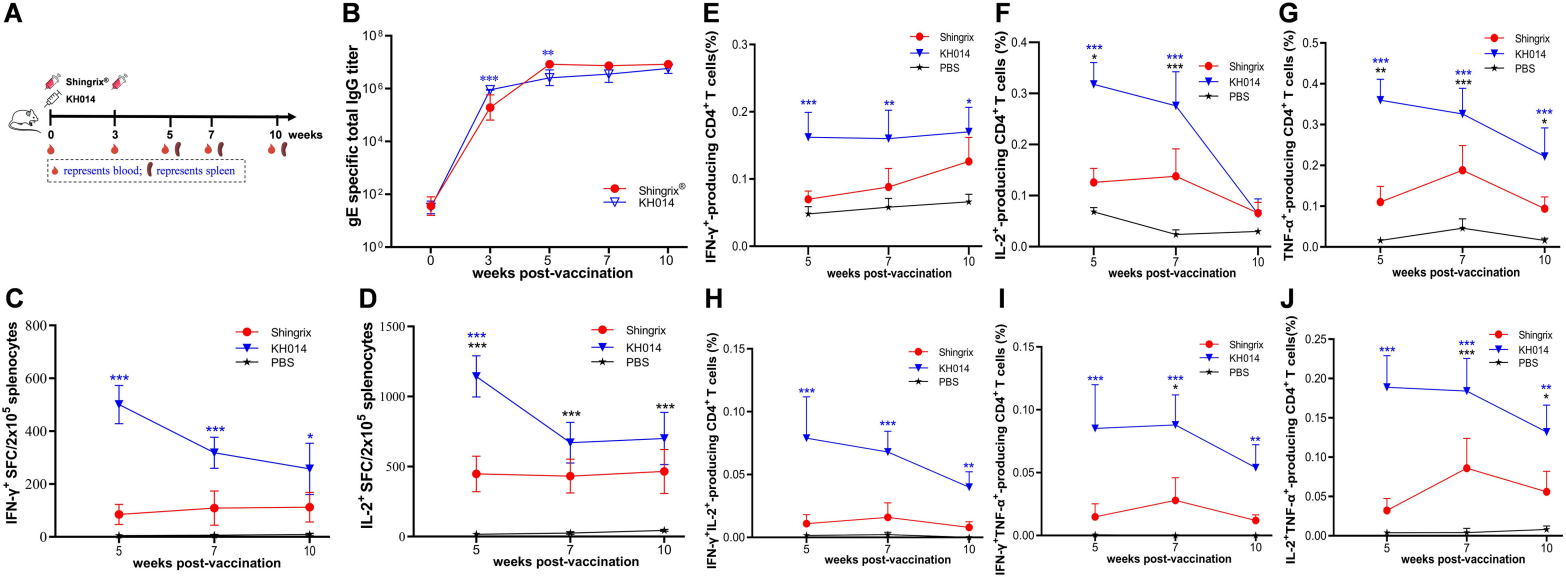
A single dose of KH014 induces long-term immunogenicity. BALB/c mice were immunized with a single-dose of KH014 mRNA vaccine (10 μg/mouse, n=5). Shingrix^®^ was included as a positive control and the same volume of PBS was injected as a placebo. Serum and splenocyte samples were collected at the indicated time points. **(A)** Scheme of immunization and sample collection schedule. **(B)** gE-specific antibody responses were determined by endpoint dilution ELISA. ELISpot assay was performed to assess IFN-γ **(C)** and IL-2 **(D)** secretion by mouse splenocytes. Percentage of antigen specific CD4^+^ T cells producing IFN-γ **(E)**, IL-2 **(F)**, TNF-α **(G)**, IFN-γIL-2 **(H),** IFN-γTNF-α **(I)**, and IL-2TNF-α **(J)** from mice splenocytes were measured by ICS assay. Data are shown as the mean ± SD. Each symbol represents one animal. *P* values were calculated by two-way ANOVA with multiple comparison tests. *P < 0.05, **P < 0.01, ***P < 0.001.

Ideal prophylactic vaccines are expected to generate long-lasting protection. To evaluate whether a single dose of the KH014 vaccine candidate could induce long-term persistence of humoral and cellular immune responses, mice were received a single intramuscular injection of 10 µg KH014. The positive control group of Shingrix^®^ was similarly designed as the previous mouse study (**Figure 5A**). Following vaccination, the body weight of mice was monitored weekly. As shown in **Supplementary Figure 4A**, mice in the Shingrix^®^ group exhibited a transient loss of body weight 7 days following booster immunization, which attribute to the high reactogenicity of Shingrix^®^ in line with clinical observation. Furthermore, several routine blood parameters and histopathological examination of major organs were assessed to evaluate the safety properties of KH014 in mice at day 35. No significant changes were observed in blood levels of routine blood parameters including white blood cell count, red blood cell count, platelet count, neutrophil count, percentage of neutrophile, percentage of monocyte, percentage of eosinophile, and percentage of eosinophil (**Supplementary Figure 4C**). The H&E staining results showed no obvious pathological changes in any of the organs (**Supplementary Figure 4B**). These results support the safety of the novel KH014 in mice.

Anti-gE binding antibodies were detected by ELISA from sera of mice collected on 0, 3, 5, 7, and 10 weeks (**Figure 5B**). Serum IgG binding antibody levels induced by a single 10 µg dose of KH014 were lower than those in Shingrix^®^ vaccinated animals at weeks 5 and 7. However, the antibody response to KH014 increased over time, reaching a level comparable to that of Shingrix^®^ by weeks 10 (**Figure 5B**). We next examined the cellular response to immunization with single dose of KH014 at 5, 7, and 10 weeks. Splenocytes from all groups were isolated and subjected to ELISpot assay (**Figure 5C, D**). We observed greater numbers of gE-specific IFN-γ/IL-2 spot-forming T cells in the KH014 group than in the Shingrix^®^ group at 5, 7, and 10 weeks. Polyfunctional T cell responses were analyzed by ICS. Similarly, flow cytometry results showed that the KH014 induced significantly higher numbers of CD4^+^ T cells across all timepoints, including gE protein-specific IFN-γ^+^ (**Figure 5E**), IL-2^+^ (**Figure 5F**), and TNF-α^+^ (**Figure 5G**) single-positive CD4^+^ T cells, as well as IFN-γ^+^IL-2^+^ (**Figure 5H**), IFN-γ^+^TNF-α^+^ (**Figure 5I**), and IL-2^+^TNF-α^+^ (**Figure 5J**) double-positive CD4^+^T cells, compared to those for the Shingrix^®^. While a statistically significant difference was observed exclusively in the frequencies of gE protein-specific IFN-γ^+^ single-positive and IFN-γ^+^TNF-α^+^ double-positive CD8^+^ T cells among stimulated splenocytes derived from KH014 immunized mice (**Supplementary Figure 5**). Altogether, these results confirm that a single dose regimen of KH014 mRNA vaccine induced superior long-term immunogenicity compared to the licensed subunit vaccine in the mouse model.

### 3.6 KH014 mRNA vaccine boosted with Shingrix^®^ produced superior cell-mediated immune

**Figure 6:**
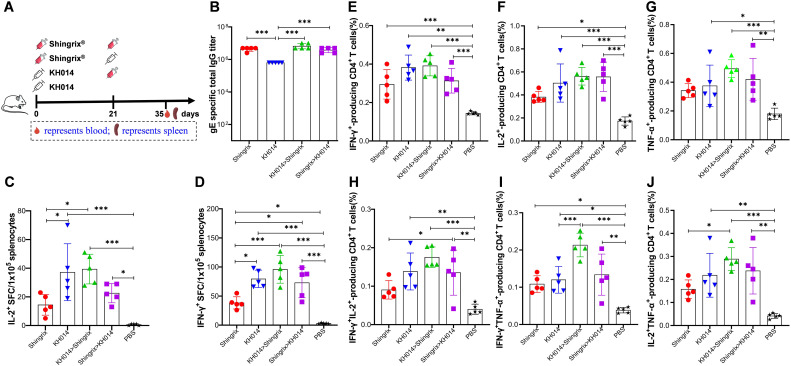
KH014 mRNA vaccine boosted with Shingrix^®^ produced superior cell-mediated immune. Groups of female BALB/c mice (n=5) received either a single-dose of KH014 mRNA vaccine (10 μg/mouse) alone, or vaccinations containing 10 μg/dose of KH014 combined with 5 μg/dose Shingrix^®^. Shingrix^®^ was included as a positive control and the same volume of PBS was injected as a placebo. Serum and splenocyte samples were collected 14 days after the boost. **(A)** Scheme of immunization and sample collection schedule. **(B)** gE-specific antibody responses were determined by endpoint dilution ELISA. ELISpot assay was performed to assess IFN-γ **(C)** and IL-2 **(D)** secretion by mouse splenocytes. Percentage of antigen specific CD4^+^ T cells producing IFN-γ **(E)**, IL-2 **(F)**, TNF-α **(G)**, IFN-γIL-2 **(H),** IFN-γTNF-α **(I)**, and IL-2TNF-α **(J)** from mice splenocytes were measured by ICS assay. Data are shown as the mean ± SD. Each symbol represents one animal. *P* values were calculated by one-way ANOVA with multiple comparison tests. *P < 0.05, **P < 0.01, ***P < 0.001.

Given the limitations of the two-dose Shingrix^®^ regimen, including systemic adverse reactions, high cost, and reduced adherence, we evaluated the humoral and cellular immune responses elicited by heterologous immunization strategies combining the KH014 mRNA vaccine and Shingrix^®^. Mice were assigned to different immunization groups: one received the conventional two-dose regimen of Shingrix^®^; another received a heterologous regimen beginning with 10 μg KH014 or 5 μg Shingrix^®^ followed by a booster (Shingrix^®^>KH014 and KH014>Shingrix^®^); and the last received only a single dose of KH014 (1xKH014). As a negative control, mice received 100 μL phosphate buffered saline (PBS) on days 0 and 21 (**Figure 6A**). We performed microscopic histopathological observation using muscle tissues at the injection sites on days 35. H&E staining of muscles at the administration site revealed slight intramuscular/perimuscular inflammatory in the Shingrix^®^ group and KH014>Shingrix^®^ groups. The PBS, 1xKH014 and Shingrix^®^>KH014 groups exhibited no inflammation at injection sites (**Supplementary Figure 6**).

ELISA results demonstrated that anti-gE IgG binding antibody titers were significantly higher in both homologous and heterologous prime-boost immunization groups compared to the single-dose KH014 group, while the IgG levels in homologous and heterologous prime-boost regimens were comparable (**Figure 6B**). To further investigate the effect of heterologous prime-boost schedules on immune response types, the cytokines (IFN-γ and IL-2) and polyfunctional T cell responses were measured using ELISpot, and flow cytometry, respectively. Compared to the two-dose Shingrix^®^ regimen, both the single-dose KH014 group and the KH014>Shingrix^®^ group exhibited a significantly higher number of gE-specific IFN-γ/IL-2 spot-forming T cells (SFCs) (**Figures 6C, D**). In contrast, priming with Shingrix^®^ followed by a KH014 booster resulted only in a slight elevation of SFCs compared to the Shingrix^®^ control. Further analysis of T-cell-mediated immunity revealed that the KH014>Shingrix^®^ group induced the highest proportions of gE-specific CD4^+^ T cells, including IFN-γ^+^, IL-2^+^, and TNF-α^+^ single-positive subsets, as well as IL-2^+^IFN-γ^+^, IL-2^+^TNF-α^+^, and IFN-γ^+^TNF-α^+^ double-positive subsets (**Figures 6E–J**). The proportions of these single- and double-cytokine-producing CD4^+^ T cells in the single-dose KH014 and Shingrix^®^>KH014 groups were comparable to those in the two-dose Shingrix^®^ control group. Frequencies of cytokine-producing CD8^+^ T cells were also assessed by flow cytometry (**Supplementary Figure 7**). The proportions of single- and double-cytokine-producing CD8^+^ T cells, except IL-2 producing CD8^+^ T cells (**Supplementary Figure 7B**), were all higher in the heterologous prime-boost immunization groups and single-dose KH014 group than that in the two-dose Shingrix^®^ control group, in which these responses were minimal or undetectable in some mice.

**Figure 7:**
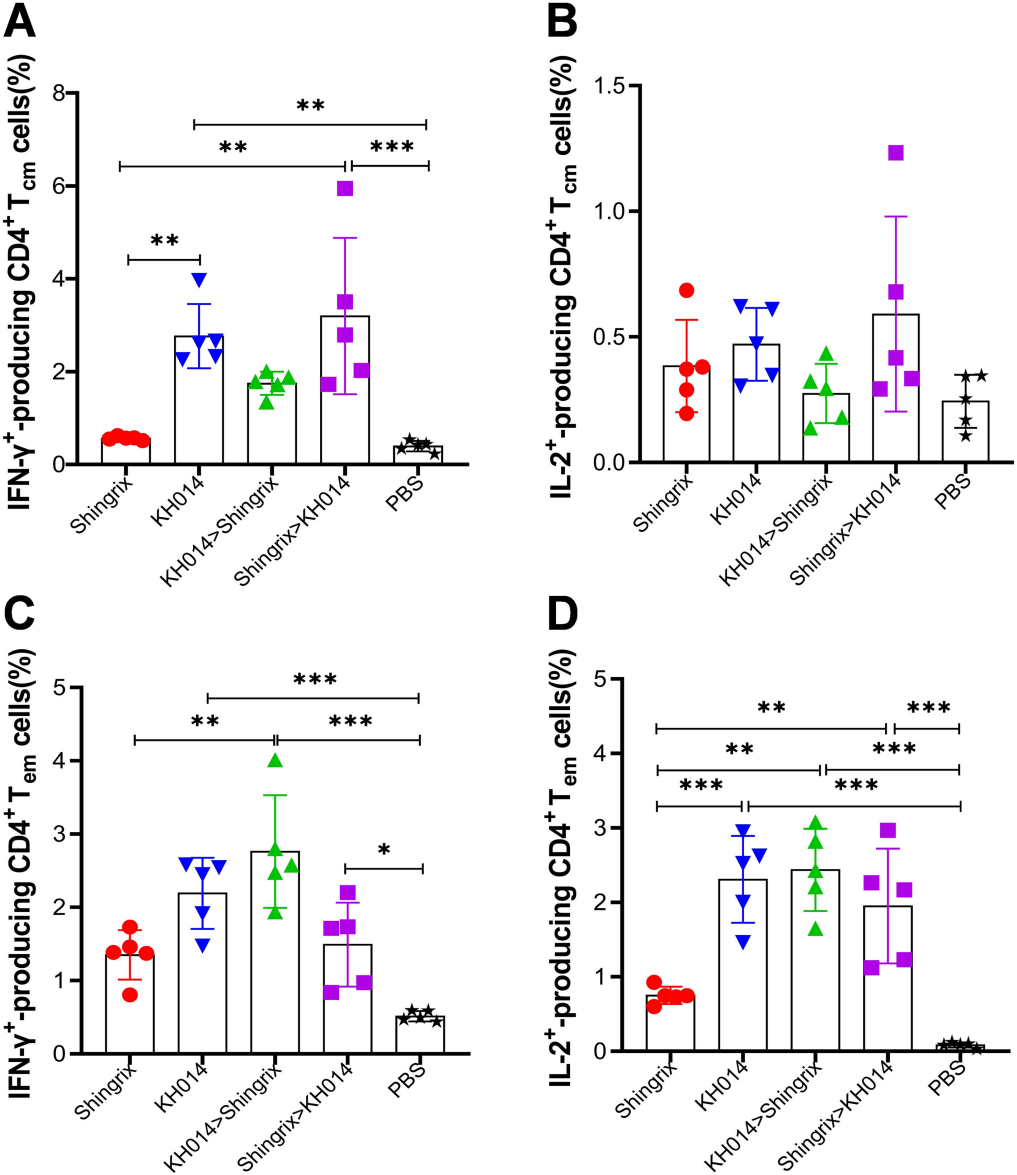
CD4^+^ memory T cells response induced by heterologous prime-boost immunization strategy. Groups of female BALB/c mice (n=5) received either a single-dose of KH014 mRNA vaccine (10 μg/mouse) alone, or vaccinations containing 10 μg/dose of KH014 combined with 5 μg/dose Shingrix^®^. Shingrix^®^ was included as a positive control and the same volume of PBS was injected as a placebo. Serum and splenocyte samples were collected 14 days after the boost. Percentage of gE specific central and effector CD4^+^ memory T cells producing IFN-γ **(A, C)** or IL-2 **(B, D)** from mice splenocytes were measured by ICS assay. Data are shown as the mean ± SD. Each symbol represents one animal. *P* values were calculated by one-way ANOVA with multiple comparison tests. *P < 0.05, **P < 0.01, ***P < 0.001.

To further evaluate a single-dose administration and heterologous prime-boost immunization strategy for inducing gE-specific memory immune response, the proportion of gE-specific CD4^+^ central memory (CD44^+^CD62L^+^) and effector memory (CD44^+^CD62L^-^) T cells induced by different immunization strategies were then assessed by flow cytometry (**Figure 7**). According to the flow cytometry analysis, a single-dose KH014 induced significantly higher frequencies of gE-specific IFN-γ-producing CD4^+^ central memory T cells (**Figure 7A**) and IL-2-producing CD4^+^ effector memory T cells (**Figure 7D**), compared to Shingrix^®^. As for the heterologous prime-boost immunization strategy, mice primed with KH014 and boosted with Shingrix^®^ were induced significantly higher frequencies of gE-specific CD4^+^ effector memory T cell response (**Figures 7C, D**), but mice in Shingrix^®^>KH014 group displayed higher proportions of gE-specific CD4^+^ central memory T cell than those in Shingrix^®^ vaccinated animals (**Figures 7A, B**). Overall, these findings highlight KH014 mRNA vaccine boosted with Shingrix^®^ strategy produced superior cell-mediated immune, and also further underscores the potential of a single-dose KH014 vaccination.

## 4 Discussion

Globally, the seroprevalence of varicella-zoster virus (VZV) among adults over age of 50 is approximately 95%, indicating that the vast majority of this population faces a lifetime risk of developing herpes zoster (HZ) (23). With the world’s population aging at an unprecedented rate, there is renewed urgency for global health action. In this context, HZ vaccination has become a critical priority to mitigate its burden on older adults (24). Two marketed vaccines are available for the prevention of HZ in China. However, limitations associated with current vaccines, such as the variable efficacy of Ganwei^®^, and the adverse reactions and adherence challenges of Shingrix^®^, draw attention to the necessity for a new-generation vaccine that is both safer and more effective. Messenger RNA (mRNA) vaccines, a representing a novel biotechnological advancement, demonstrate distinct advantages in combating both newly emerging, unknown pathogens and highly virulent infectious agents (25). Herein, we developed a modified mRNA-based vaccine candidate, designated KH014, which encodes the VZV gE protein and is encapsulated in lipid nanoparticles. A single immunization with KH014 elicited a more robust immunogenic response than the commercially available Shingrix^®^ vaccine, and demonstrated sustained long-term immunity with a favorable safety profile.

The VZV glycoprotein E (gE) protein is expressed not only on the surface of viral particles and infected cells but also plays a critical role in viral transmission and infection (26). Preclinical and clinical evidence has established the full-length gE as an ideal antigen for eliciting robust humoral and cellular immune responses. The gE protein is localized to both the plasma membrane and the cytoplasm of VZV-infected cells, where it is presumed to be present in the membranes of intracellular vesicles or organelles (27). Structurally, the full-length of gE consists of four primary domains including a signal peptide located at the N-terminal end, followed by an ectodomain, a transmembrane domain and finally a cytoplasmic tail at the C-terminal end (15, 28). Recently, Munoz-Moreno et al. compared several gE-based mRNA vaccine constructs including truncated, soluble and epitope-enriched variants, and confirm that the full-length gE construct induced the highest gE-specific IgG titers and most robust T cell responses (11). In our study, the KH014 candidate vaccine was developed based on an optimized full-length gE antigen and encapsulated within lipid nanoparticles (LNPs), enabling lyophilized storage at 2-8 °C under ambient conditions (29). A limitation of this study is the lack of a direct head-to-head comparison between its antigen design and the truncated gE mRNA control group within the same experimental framework. Thus, it remains unclear whether the observed immunological profile is specifically attributable to the full-length antigen design or to intrinsic advantages of the mRNA-LNP platform, such as its self-adjuvanting properties … However, our antigen design is strongly supported by previous studies involving direct comparisons of gE variants (11, 17). This suggests that the full-length antigen may contribute to the quality of immune responses beyond the delivery platform itself, potentially by preserving key conformational epitopes and ensuring correct cellular localization. Future research incorporating direct comparisons of different antigen designs would be valuable for further optimizing VZV mRNA vaccine candidates. Nevertheless, the primary objective of this work was to evaluate novel single-dose regimens and heterologous prime-boost strategies using our lead candidate KH014, and the results presented here provide a robust foundation for its subsequent development.

Although evidence indicates that VZV-specific antibodies are dispensable for protection, they play a role in controlling both primary infection and reactivation (30). Clinical data from hemodialysis patients who received recombinant VZV vaccine showed higher VZV IgG levels after one year compared to those who did not, supporting the efficacy of VZV-specific antibodies in this vulnerable patient population (31). We measured the anti-gE IgG elicited by KH014 and found that the two-dose administration of KH014 induced a comparable level of gE-specific IgG to Shingrix^®^, while a single dose yielded significantly lower levels, a finding that potentially links the reduced efficacy to lower antigen expression in the single-dose regimen. Notably, ten weeks after administration, a single 10 µg dose of KH014 induced gE-specific antibody titers that were comparable to those induced by Shingrix^®^, pointing to a potential advantage of KH014 in the long-term durability of the humoral response, which is closely associated with memory B cell activity (32). When comparing immune responses across different vaccine platforms, the basis for dose selection warrants careful consideration. The optimal immunogenic dose for an mRNA-LNP vaccine cannot be directly equated, on a simple microgram basis, with that of an adjuvanted recombinant protein vaccine. This is due to fundamental distinctions in how antigens are produced, presented to the immune system, and accompanied by innate immune stimulation. In this study, the dose ranges were chosen based on established preclinical models for each platform to enable a functionally relevant comparison. This article highlights that a single, relatively low dose of KH014 elicits a cellular immune response superior to that induced by two doses of protein subunit vaccines. This finding underscores the inherent advantage of the mRNA-LNP platform in efficiently driving potent T-cell responses. The observed immunological advantages likely reflect a qualitative difference in platform technology rather than a mere quantitative difference in antigen amount. A previous study concluded that a gE mRNA vaccine likely induces a more sustained response than Shingrix^®^, as evidenced by its induction of significantly higher levels of gE-specific memory B cells, which correlated well with antibody responses (16). Neutralizing antibody titers are important for the varicella vaccine and it also has a role in protecting against VZV reactivation via neutralizing cell-free virus and inhibiting cell-to-cell spread (21, 30, 33). We employed *in vitro* microneutralization assay to investigate neutralizing antibody titers and found our KH014 induced significantly superior neutralizing antibody titers, potentially indicating an enhanced capacity to block viral entry and spread. It is well-documented that the increase in herpes zoster (HZ) risk with age is primarily driven by the age-related decline in VZV-specific T-cell immunity, rather than humoral immunity (34, 35). Therefore, neutralizing antibody titer was not considered a key indicator and was not measured in follow-up experiments.

As a key component of T-cell-mediated immunity, antigen-specific CD4^+^ T cells are essential for protecting the host against the reactivation of latent VZV (2, 30). Type 1 cytokines (IL-2 and IFN-γ) function to promote cellular immunity by enhancing macrophage cytotoxicity, thereby combating intracellular pathogens (36). In this study, KH014 induced higher levels of type 1 cytokines than Shingrix^®^ following both single and two-dose vaccination regimens, indicating a strong Th1-biased response from the gE mRNA vaccine. This finding aligns with those reported for previous VZV mRNA vaccines. Furthermore, we observed robust induction of CD4^+^ T cells response upon administration of a single or two doses of KH014, implying that KH014 generated a potent CD4^+^ T cell response in an antigen specific manner. Polyfunctional T cells, defined by their ability to co-express multiple cytokines or other effector molecules, have been correlated with protective immunity and improved clinical outcomes in numerous infectious diseases (37, 38).Data from the polyfunctional CD4^+^ T cell response illustrate that a greater proportion of mono- and bi-functional cells in the KH014 group compared to the Shingrix^®^ group, in line with the findings of previous VZV mRNAs. Furthermore, beyond CD4^+^ T cell responses, recent evidence highlights the critical role of CD8^+^ T cells in controlling viral replication in the ganglia during active HZ. In this context, our vaccine candidate KH014 induced a superior CD8^+^ T cell response compared to Shingrix^®^, which elicited only low or undetectable levels of bifunctional cells in a subset of animals. These findings are consistent with previous studies showing that Shingrix^®^ does not elicit CD8^+^ T cell responses in mouse models or humans, whereas mRNA vaccines effectively activate them (39). This difference in CD8^+^ T cell responses across vaccine platforms may be attributed to the fact that mRNA vaccines enable intracellular antigen expression, thereby activating innate immune pathways and leading to efficient CD8^+^ T cell responses through MHC class I presentation (11). It is noteworthy that during HZ infection, the upregulation of MHC molecules expression in human ganglia indicates an active role of these cells in orchestrating immune responses (40). More importantly, we observed that the CD4^+^ memory T cell responses (including central and effector memory T cells) elicited by KH014 were superior to those elicited by Shingrix^®^. This implies that KH014 has an enhanced ability to induce long-lasting immunity against VZV, a conclusion later supported by subsequent studies on long-term CD4^+^ T cell responses (17).The inherent instability of RNA shortens the *in vivo* duration of action of mRNA vaccines, which may in turn hinder the development of single-dose vaccination regimens (41). Herein, we assessed the feasibility of a single-dose KH014 regimen for the first time and observed that a single dose of the KH014 regimen elicited superior VZV-specific cell-mediated immunity, particularly in Th1 CD4^+^ T cell responses, compared to Shingrix^®^. Interestingly, our data demonstrate a single-dose KH014 dosages above 10 μg result in only minor increase cellular immune responses, which was similar with a previous study (10), suggesting dosages above this range will not translate into additional immunological benefit. Therefore, a subsequent study was conducted using a single 10 µg dose of KH014 to investigate the long-term persistence of both humoral and cellular immune responses. With regard to the durability of IgG antibody titers, although the Shingrix^®^ group initially had higher antibody IgG titers than the single-dose 10 μg KH014 group, the titers in both groups plateaued after 7 weeks and thereafter maintained comparable levels. Unlike IgG antibody titers, a single 10-μg dose of KH014 induced significantly higher Type 1 cytokine levels than Shingrix^®^ initially, while it subsequently exhibited a sharper decline, stabilizing at a level only marginally higher. This observation recapitulated the pattern of responses reported in a previous study (10), suggesting that a single administration of KH014 can induce an immunogenicity level comparable to Shingrix^®^. However, a limitation of this study is the absence of data on vaccine-induced longitudinal neutralizing antibody responses, on which a recent study has confirmed a significant concordance with gE-specific cellular immunity (20).

Finally, the immune efficacies of heterogeneous prime-boost immunization strategies with KH014 and Shingrix^®^ were evaluated. We observed that the heterologous KH014/Shingrix^®^ prime/boost vaccine strategy induced stronger humoral and cellular immune responses than either KH014 or Shingrix^®^ alone. Interestingly, the heterologous KH014/Shingrix^®^ prime/boost vaccine strategy elicited stronger immune efficacies than the Shingrix^®^ prime/KH014 boost vaccine strategy, a similar phenomenon observed with heterologous immunization against SARS-CoV-2 (42), influenza virus (43), and HIV (44). This indicates that the order in which the vaccines are administered play a key role in establishing immunogenicity. Mounting evidence points to heterologous sequential immunization as a strategy that enhances protective potency and breadth by harnessing complementary immune mechanisms: mRNA priming to shape T-helper cell bias and protein-based subunit vaccines to evoke strong antibody responses (43, 45). Consequently, our study provides a framework for developing heterologous sequential immunization strategies with improved efficacy and breadth of protection. In addition, consistent with our above findings, we again demonstrated that a single 10-μg dose induced superior humoral and cellular immune responses compared to the Shingrix^®^ control group, suggesting the feasibility of a single-dose immunization regimen.

This study has certain limitations that need to be addressed in future work. The ideal approach to validate the protective efficacy of KH014 would involve a viral challenge model. However, due to the lack of adequate animal models that fully recapitulate VZV reactivation in humans (46), the protective efficacy of our KH014 vaccine cannot be definitively ascertained, despite its ability to induce robust immunogenicity in mice. The current experimental design does not allow a clear separation between immunological effects driven by the KH014 platform itself and those arising from non-equivalent dosing schedules or dose levels. It remains to be further validated whether the superior immunogenicity induced by KH014, compared with Shingrix, is attributable to the mRNA technology platform or to differences in the dosing regimen and dosage. Furthermore, future studies will assess gE protein expression in the draining lymph nodes following KH014 to provide direct visualization of antigen expression and further elucidate the *in vivo* kinetics of our vaccine candidate. To summarize, we developed a novel VZV mRNA vaccine candidate, KH014, which demonstrated superior immunogenicity to the licensed vaccine Shingrix^®^ in mice. Notably, a single dose of KH014 was sufficient to elicit robust VZV-specific cell-mediated immunity and long-term immune responses. To our knowledge, this is the first study to demonstrate that a single-dose VZV mRNA vaccine can induce both potent humoral and cellular immunity, highlighting the feasibility of a single-dose immunization strategy. Finally, we investigated the optimal immune strategy of VZV mRNA vaccine and protein-subunit vaccine in mice and found that the cell immune response elicited by heterologous prime-boost regimen was equal with a single dose of mRNA vaccine, but stronger than Shingrix^®^. These findings provide valuable information to the design and optimization of VZV vaccines, which supports the development of broader varicella vaccination strategies in the future.

## Data Availability

The original contributions presented in the study are included in the article/**Supplementary Material**. Further inquiries can be directed to the corresponding author.
