# Supplementary material for: A single-dose mRNA vaccine induces potent and long-lasting humoral and cellular immunity against the varicella-zoster virus in a murine model
Source: Front Immunol. 2026 Mar 23;17:1771359. doi: 10.3389/fimmu.2026.1771359 (PMC13051512; doi:10.3389/fimmu.2026.1771359)
Supplement: Supplementary file 1 [file Table1.docx]

**A single-dose mRNA vaccine induces potent and long-lasting humoral and cellular immunity against the varicella-zoster virus**

**Supplementary Figures**


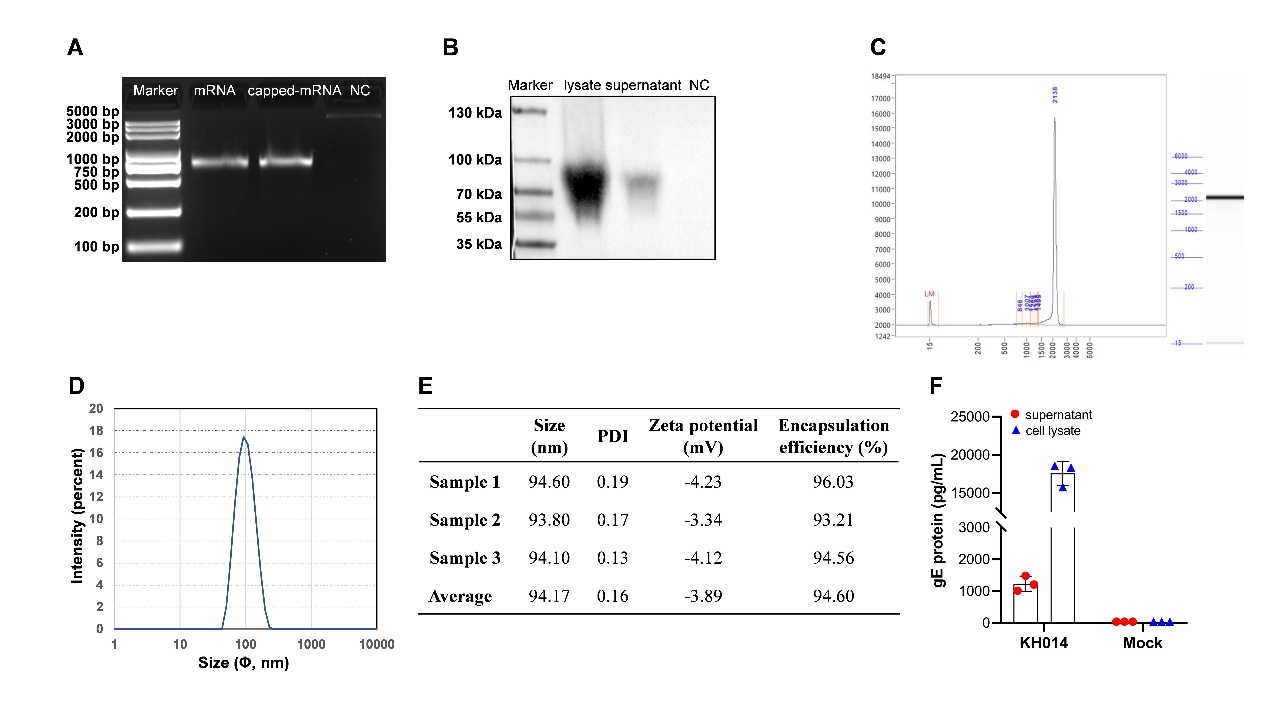


**Supplementary Figure S1**. Characterization of KH014 mRNA vaccine. **(A)** Electrophoresis showing the integrity and purity of synthesized gE mRNA. **(B)** Western blot of cell supernatants and cell extracts after transfecting HepG2 cells with KH014 mRNA, exhibiting a clear band at 55-100 kDa. **(C)** Liquid capillary electropherograms of *in vitro*-transcribed KH014 mRNA. Peaks represent KH014 mRNA (2100 nt). **(D)** Representative intensity-size graph of the redissolved KH014 measured by dynamic light-scattering method. **(E)** Table summarizing LNP physicochemical aspects including polydispersity index (PDI), zeta-potential, and encapsulation rate. **(F)** *In vitro* expression of KH014 mRNA-LNP in cell lysate and supernatant was assessed by ELISA.


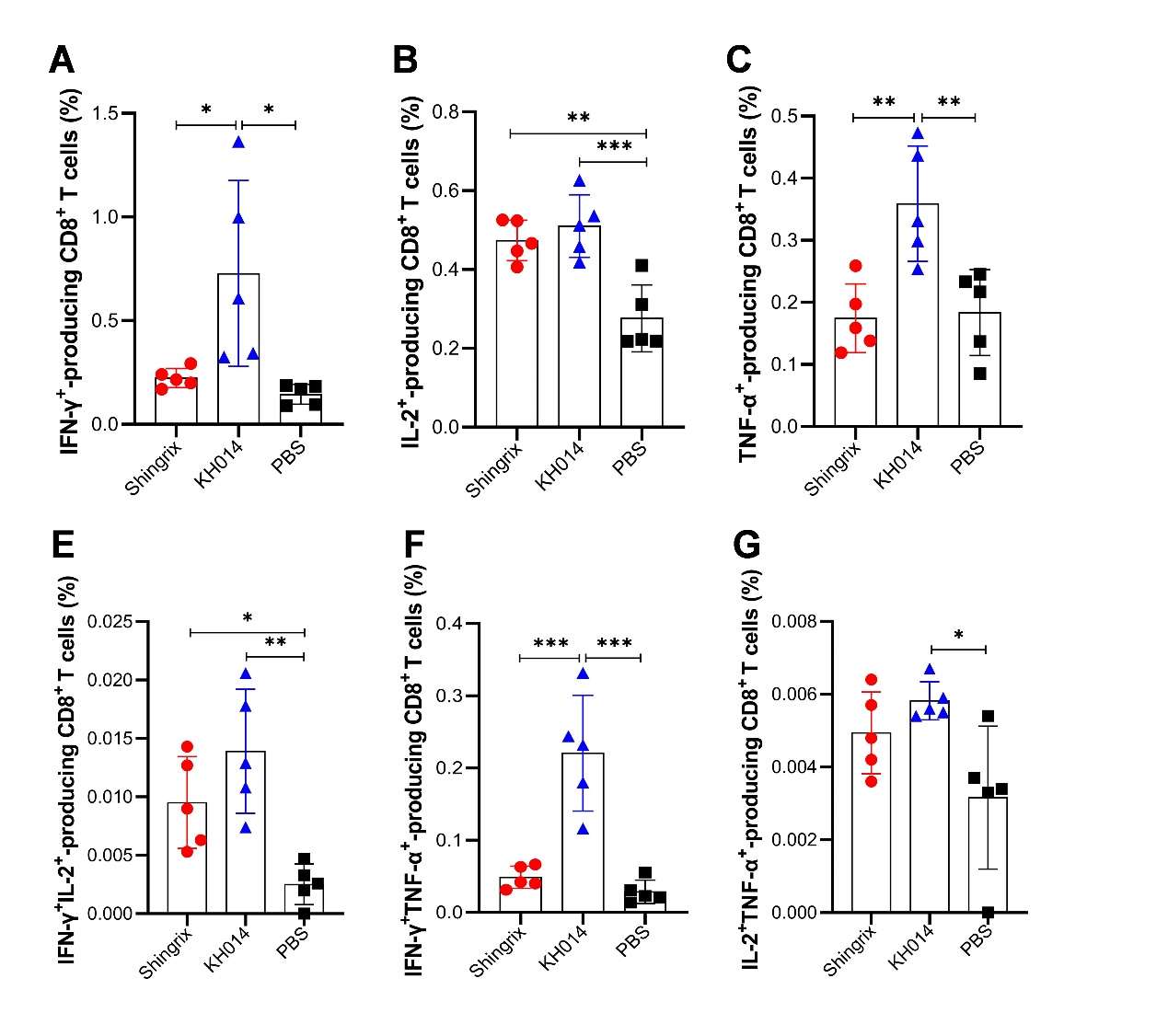
 **Supplementary Figure S2**. CD8^+^ T cell response induced by KH014 mRNA vaccine. Female BALB/c mice (n=5) received two doses of either KH014 mRNA vaccine, Shingrix^®^, or PBS, administered via intramuscular injection. Percentage of antigen specific CD8^+^ T cells producing IFN-γ **(A)**, IL-2 **(B)**, TNF-α **(C)**, IFN-γIL-2 **(D),** IFN-γTNF-α **(E)**, and IL-2TNF-α **(F)** from mice splenocytes were measured by intracellular cytokine staining (ICS) assay on day 35 (n=5). One-way Analysis of Variance (ANOVA) with Tukey’s multiple comparison test was performed to determine statistical significance. **p* < 0.05, ***p* < 0.01, ****p* < 0.001.


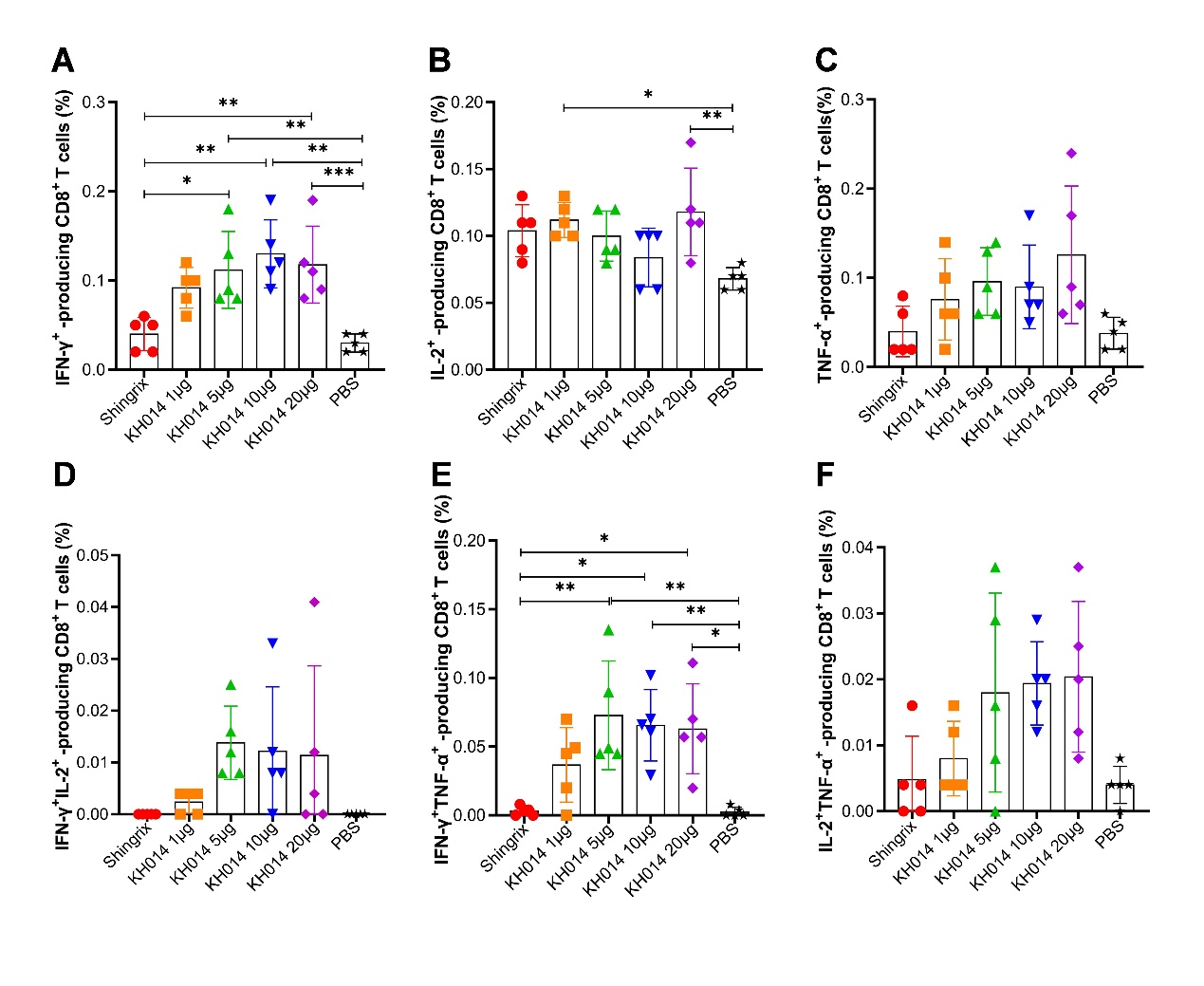
 **Supplementary Figure S3**. CD8^+^ T cell response induced by a single-dose administration of KH014 mRNA vaccine. Groups of female BALB/c mice (n=5) received a single administration of KH014 mRNA at different dosage levels (1, 5, 10, and 20 μg). Shingrix^®^ and PBS, administered via intramuscular injection with two-dose, used as positive and negative control, respectively. Percentage of gE specific CD8^+^ T cells producing IFN-γ**(A)**, IL-2 **(B)**, TNF-α**(C)**, IFN-γIL-2 **(D),** IFN-γTNF-α**(E)**, and IL-2TNF-α **(F)** from mice splenocytes were measured by intracellular cytokine staining (ICS) assay on day 35 (n=5). One-way Analysis of Variance (ANOVA) with Tukey’s multiple comparison test was performed to determine statistical significance. **p* < 0.05, ***p* < 0.01, ****p* < 0.001.


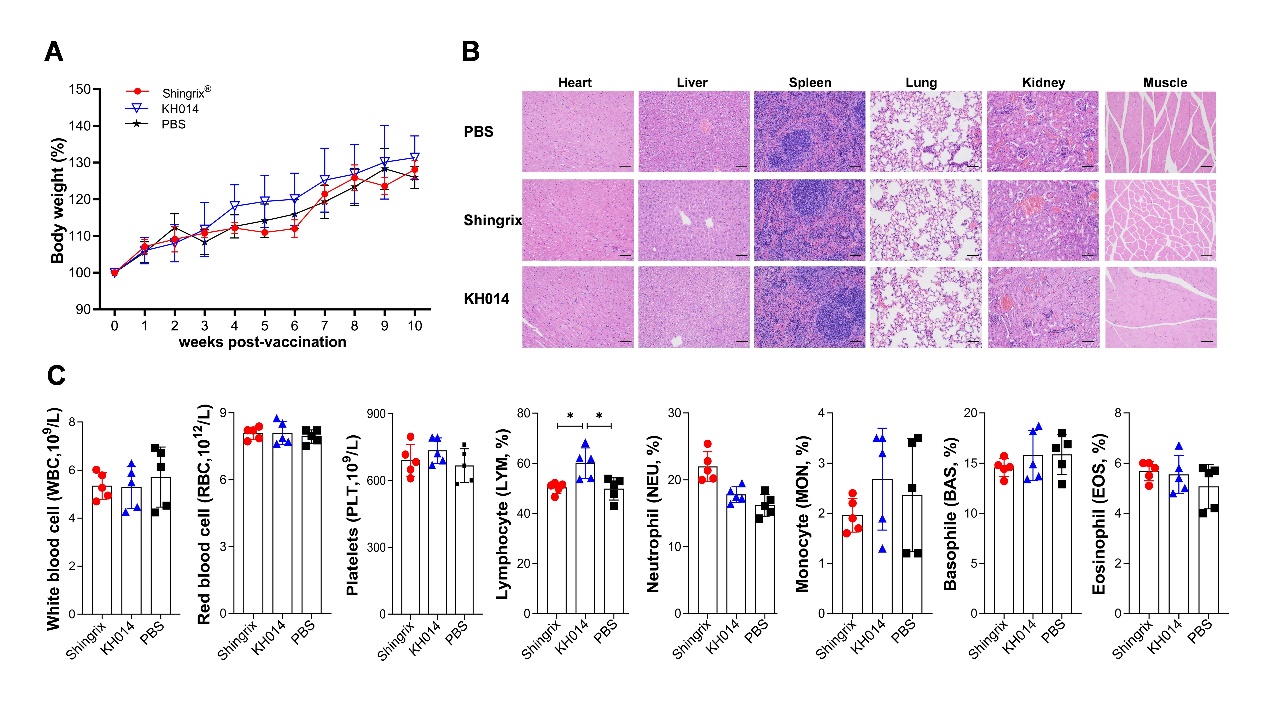


**Supplementary Figure S4**. Safety evaluation of KH014 mRNA vaccine in mice. Each mouse received a single-dose 10 µg KH014 mRNA vaccine. As a positive control, mice received two intramuscular 5-μg doses of Shingrix^®^ on days 0 and 21. **(A)** The body weight of mice was monitored weekly during 10 weeks after immunization. **(B)** Histopathological examination (H&E) of the heart, liver, spleen, lung, kidney, and muscle in BALB/c mice on day 35 after immunized with a single-dose 10 µg KH014 mRNA vaccine, two intramuscular 5-μg doses of Shingrix^®^ or the same volume of PBS. **(C)** Levels of haematology parameters on day 35 were measured. One-way Analysis of Variance (ANOVA) with Tukey’s multiple comparison test was performed to determine statistical significance. **p* < 0.05.


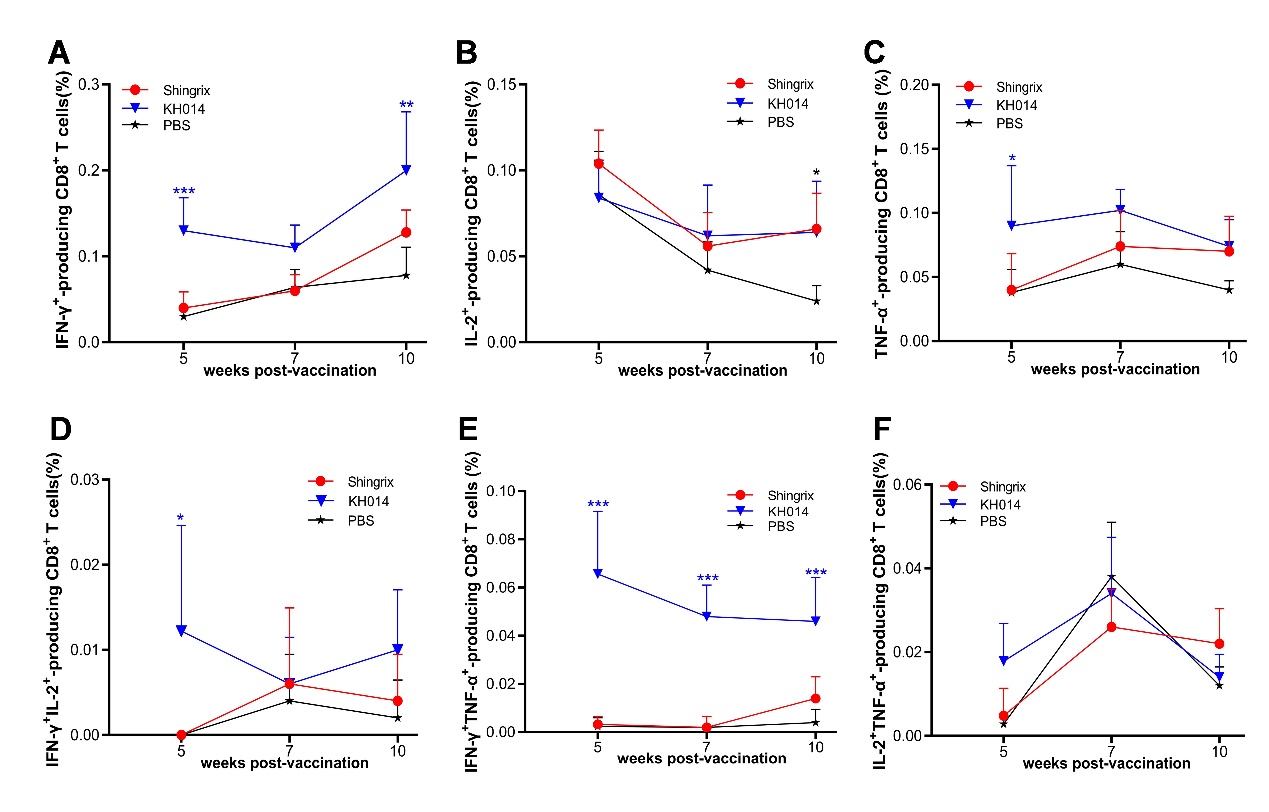
 **Supplementary Figure S5**. Duration of CD8^+^ T cell response induced by KH014 mRNA vaccine. BALB/c mice were immunized with a single-dose of KH014 mRNA vaccine (10 μg/mouse, n=5). Shingrix^®^ was included as a positive control and the same volume of PBS was injected as a placebo. Percentage of gE specific CD8^+^ T cells producing IFN-γ**(A)**, IL-2 **(B)**, TNF-α**(C)**, IFN-γIL-2 **(D),** IFN-γTNF-α**(E)**, and IL-2TNF-α **(F)** from mice splenocytes were measured by intracellular cytokine staining (ICS) assay at 5, 7, and 10 weeks. Two-way Analysis of Variance (ANOVA) with Tukey’s multiple comparison test was performed to determine statistical significance. **p* < 0.05, ***p* < 0.01, ****p* < 0.001.


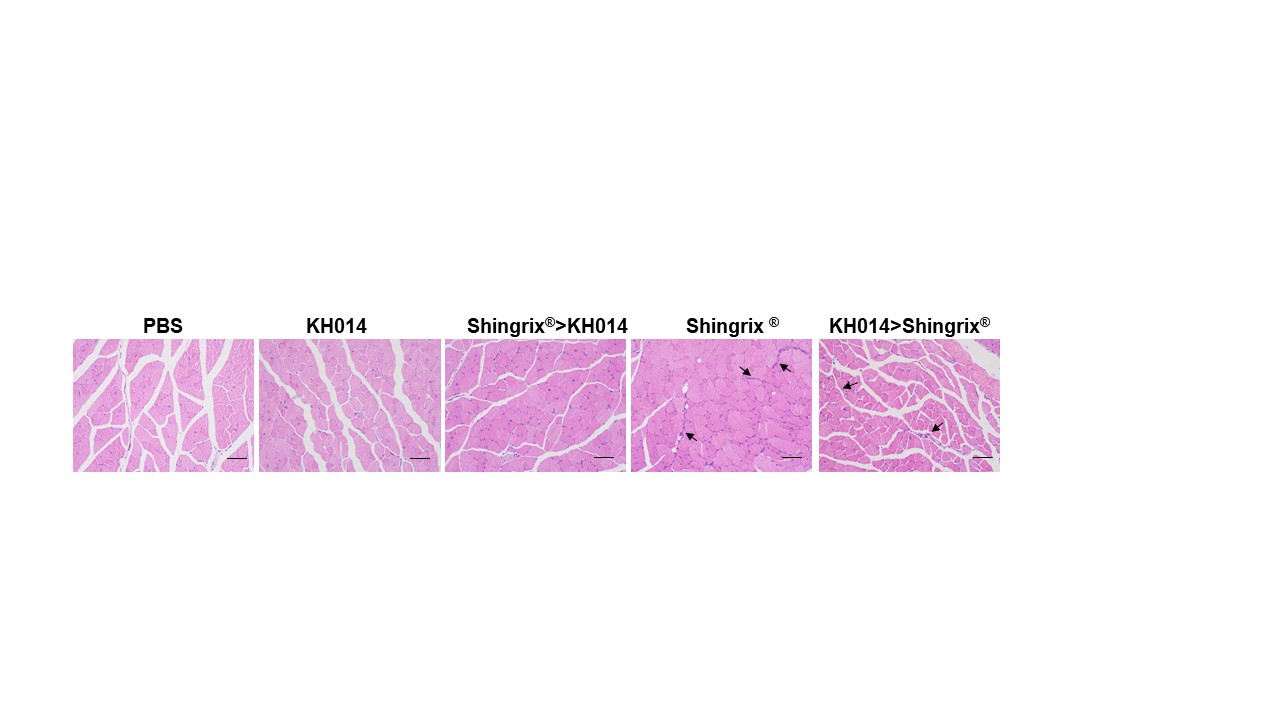


**Supplementary Figure S6**. Safety evaluation of heterologous immunization strategies. H&E staining of muscles at the administration site on days 35. Black arrows indicate inflammation.

**Supplementary Figure S5**.


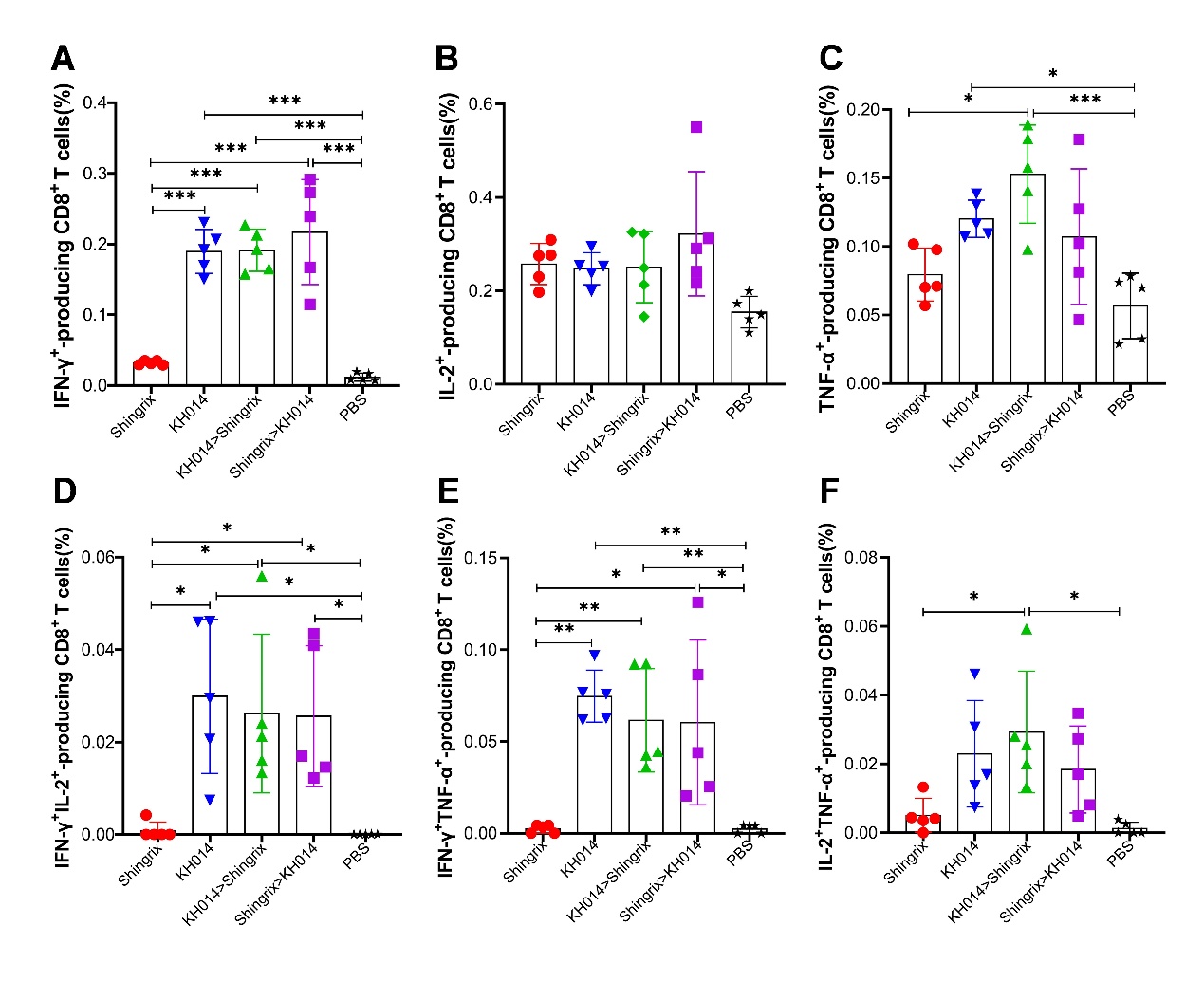
 **Supplementary Figure S7**. CD8^+^ T cell response induced by heterologous prime-boost immunization strategy. Groups of female BALB/c mice (n=5) received either a single-dose of KH014 mRNA vaccine (10 μg/mouse) alone, or vaccinations containing 10 μg/dose of KH014 combined with 5 μg/dose Shingrix^®^. Shingrix^®^ was included as a positive control and the same volume of PBS was injected as a placebo. Percentage of gE specific CD8^+^ T cells producing IFN-γ**(A)**, IL-2 **(B)**, TNF-α**(C)**, IFN-γIL-2 **(D),** IFN-γTNF-α**(E)**, and IL-2TNF-α **(F)** from mice splenocytes were measured by intracellular cytokine staining (ICS) assay on day 35. One-way Analysis of Variance (ANOVA) with Tukey’s multiple comparison test was performed to determine statistical significance. **p* < 0.05, ***p* < 0.01, ****p* < 0.001.
